# Systemic Lymphoma Masquerading as Multiple Sclerosis Relapse

**DOI:** 10.7759/cureus.48432

**Published:** 2023-11-07

**Authors:** Lisle W Blackbourn, Tiffani S Franada, Sarah E Bach

**Affiliations:** 1 Neurology, University of Illinois College of Medicine Peoria, Peoria, USA; 2 Pathology, University of Illinois College of Medicine Peoria, Peoria, USA

**Keywords:** enhancing brain lesions, fingolimod, multiple sclerosis flare-ups, multiple sclerosis, lymphoma

## Abstract

Multiple sclerosis (MS) is a chronic immune-mediated central nervous system disease that can affect both the brain and spinal cord. Given that MS can occur at any location in the brain or spinal cord and can lead to a variety of symptoms, this can lead to difficulty in diagnosing MS versus other conditions mimicking MS.

Here we present a case of a 69-year-old female with a history of relapsing-remitting MS diagnosed in 2002 and melanoma status post-excision who exhibited progressive neurological decline over eight weeks characterized by right internuclear ophthalmoplegia, bilateral ataxia, and left hemiparesis sparing the face. Mimics of MS can include various inflammatory, neoplastic, infectious, metabolic, and genetic disorders. The diagnosis of MS-mimicking diseases can be especially challenging for someone with a known history of MS. A biopsy should be considered for new lesions seen on imaging if acute immunotherapies have no response to the clinical patient’s symptoms.

Given the wide variety of symptoms that can present with MS, it is important to keep a broad range of differential diagnoses when considering MS, even in those with a known history of MS.

## Introduction

Multiple sclerosis (MS) is a chronic immune-mediated central nervous system disease that can affect both the brain and spinal cord [1]. Multiple sclerosis is one of the most common neurological disabling disorders in young adults. It is diagnosed using McDonald's diagnostic criteria to show the dissemination in time and space of lesions in the central nervous system, with symptoms varying depending on the lesion locations [2, 3]. Imaging techniques such as MRI are a crucial diagnostic tool, with serology, cerebrospinal fluid (CSF) analysis, and electrophysiology studies all aiding in the diagnosis of MS [4]. This can, however, lead to a difficult diagnosis and allow for other conditions to mimic MS [5]. These can include various inflammatory, neoplastic, infectious, metabolic, and genetic disorders. Here we present a case of a 69-year-old female patient with diagnosed MS having new-onset systemic lymphoma masquerading as relapse of MS.

## Case presentation

The patient was a 69-year-old female with a history of relapsing-remitting MS diagnosed in 2002 at age 48 and melanoma status post-excision who exhibited progressive neurological decline over eight weeks characterized by right internuclear ophthalmoplegia, bilateral ataxia, and left hemiparesis sparing the face. When the patient was initially diagnosed with MS, she presented with symptoms of diplopia, incoordination, and motor fatigue in the hands that lasted a few days. At the time, the patient had an MRI displaying lesions and had a lumbar puncture with oligoclonal bands present. She tested positive for human polyomavirus 2 (John Cunningham/JC virus) with a serum titer of 1.07 in 2018. She was treated with interferon beta-1b initially for nine months but stopped due to developing neutralizing antibodies. She then transitioned to glatiramer acetate, which she took for seven years before stopping for injection-related reactions, and she then took fingolimod for the past nine years. While on these medications, she had a stable clinical and radiologic course, with a baseline Expanded Disability Status Scale (EDSS) of 1.5.

The patient presented to the hospital with complaints of worsening balance and tripping with a likely left foot drop. The patient was also experiencing urinary incontinence. The MRI of the brain showed a severe burden of demyelinating plaques in the right cerebellar and bilateral hemispheric white matter, consistent with the patient's history of MS. There were also multiple enhancing plaques showing restricted diffusion in the left occipital, right parietal, and right frontal periventricular white matter, as well as along the right corpus callosum, consistent with active demyelination. The MRI of the cervical spine also showed vague areas of increased signal consistent with MS. Labs showed leukocytosis with a white blood cell count of 14.12 10(3)/mcL and an absolute lymphocyte count of 0.52 10(3)/mcL. She also tested positive for COVID-19. She received a five-day course of IV methylprednisolone, followed by two weeks of oral prednisone. The patient's symptoms, however, persisted, and she declined neurologically. She then returned to the MS clinic, where she was directly admitted to the hospital.

**Figure 1:**
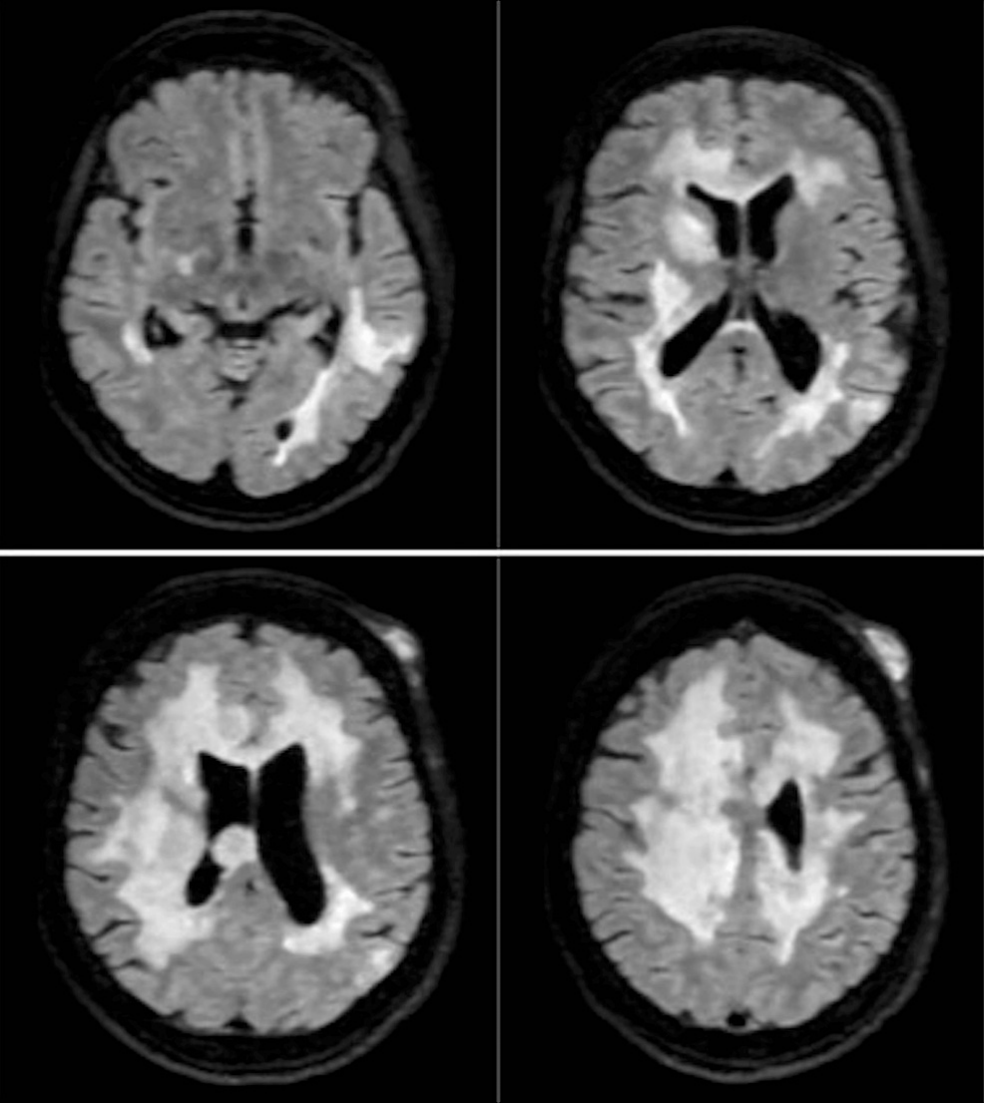
Axial view of MRI T2 FLAIR sequencing showing mass lesions within the supratentorial region bilaterally FLAIR: fluid-attenuated inversion recovery

**Figure 2:**
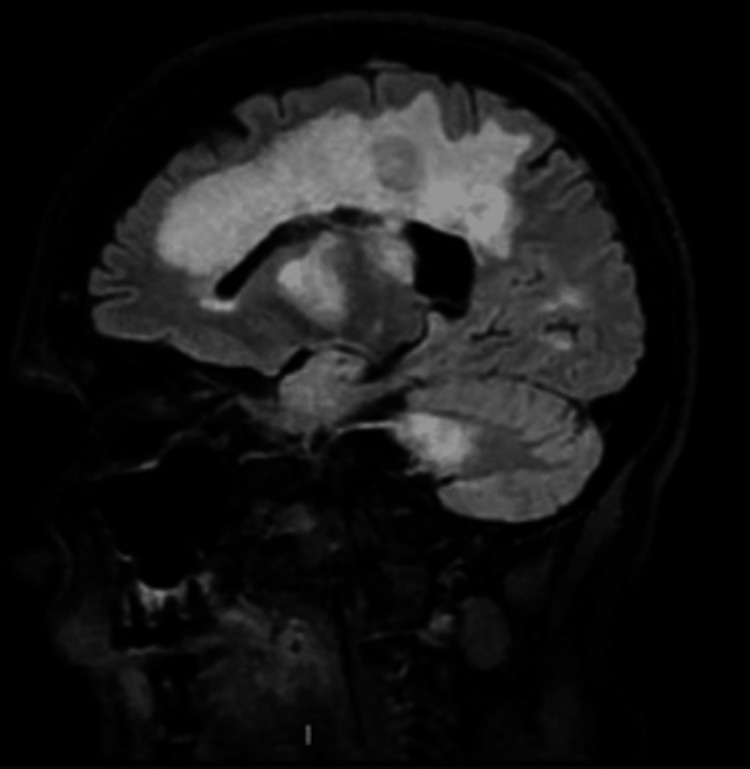
Sagittal view of MRI T2 FLAIR sequence of the lesions FLAIR: fluid-attenuated inversion recovery

On a physical exam upon hospital admission, the patient had right internuclear ophthalmoplegia with left hemiparesis sparing the face. Left lower and upper extremity weakness was rated as a 2/5 in all muscle groups. The patient had intact sensation throughout and 3+ reflexes in the bilateral biceps, triceps, brachioradialis, and patellar and Achilles tendons. Hoffman’s reflex was negative, toes deferred bilaterally, and severe ataxia was seen in all limbs. The patient also complained of a new red abdominal skin mass that developed one week before admission. Differential diagnoses at the time included tumefactive multiple sclerosis, lymphoreticular disease, metastatic disease, and progressive multifocal leukoencephalopathy (PML). The patient was started on high-dose steroids, which resulted in steroid-induced psychosis. Her mentation returned to baseline after steroid discontinuation. A five-day course of plasmapheresis was completed with improvement in left lower extremity weakness. A lumbar puncture showed normal cytology, glucose, cell count and differential, negative gram stain, and elevated protein. The JC virus was not detected in the CSF via a polymerase chain reaction (PCR) test. Repeat MRI showed progression of multiple enhancing lesions, as seen in Figures 1-2.

**Figure 3:**
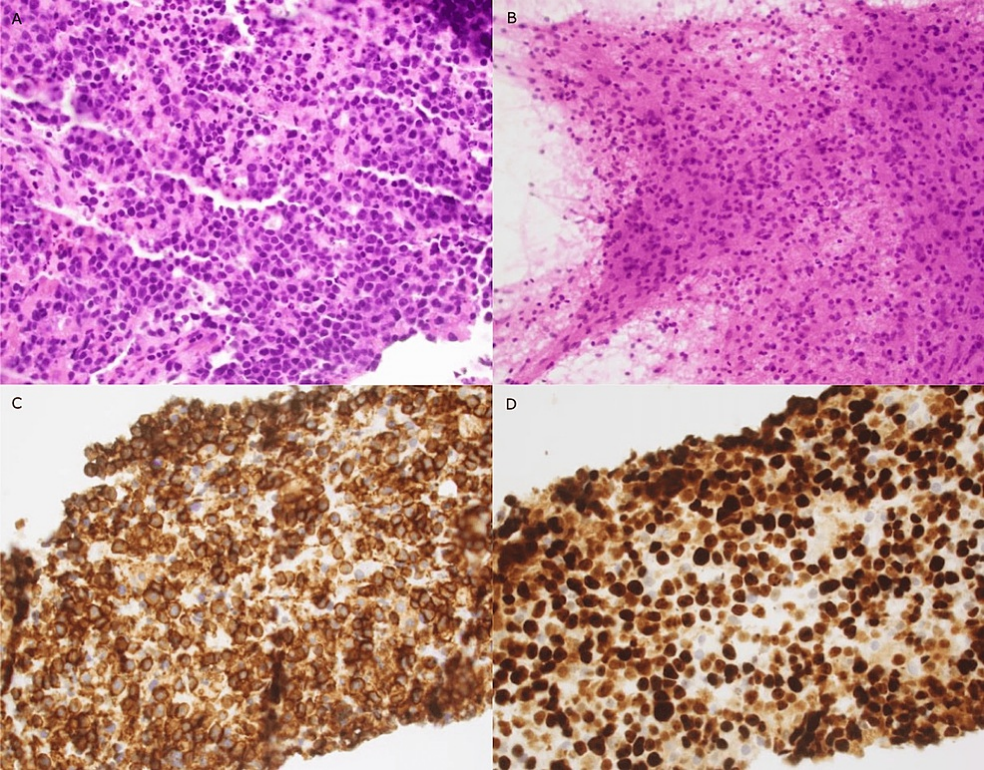
Histology and immunostaining of the brain tissue sample: A) hematoxylin and eosin stain (H&E); B) smear H&E C) CD20 staining; D) Ki67 staining.

The neurosurgery department was consulted to perform a brain biopsy of the right frontal lesion, which resulted in aggressive B-cell lymphoma. Histology showed an infiltrate of medium- to large-sized atypical lymphoid cells, which displayed ovoid to slightly angulated or irregular hyperchromatic nuclei with prominent nucleoli and scant cytoplasm. There was also moderate nuclear pleomorphism and numerous apoptotic bodies were identified. Histology and immunostaining results from the sample can be seen in Figure 3 and Table 1.

**Table 1:** Immunostaining results performed on the brain biopsy tissue with positive markers seen typically in B-cell lymphoma and negative for T-cell markers. GFAP: glial fibrillary acidic protein; +: positive; -: negative

Immunostaining Results					
Tested	Result	Tested	Result	Tested	Result
CD3	-	BCL1	-	PAX5	+
CD5	+	BCL2	+	Pancytokeratin	-
CD10	-	BCL6	+ in subset < 30%	Synaptophysin	-
CD20	+	MUM1	+ in subset > 30%	GFAP	-
CD23	-	C-myc	+ in subset 25 to 30%	Epstein-Barr virus	-
CD30	-	Ki-67	+ in very high subset 90%		

Flow cytometry analysis from the sample revealed a CD5+ monoclonal B-cell population that was positive for CD 19, CD 20, CD 45, CD200, PAX5, and dim surface lambda light chain and negative for other markers tested. The Ki-67 marker was very high at 90%. An abdominal wall biopsy also resulted in aggressive, mature B-cell lymphoma. The patient at this time decided not to pursue further treatment.

## Discussion

The wide variety of symptoms that can be seen in MS can lead to a difficult diagnosis and allow for other conditions to mimic MS. This can be especially challenging for someone with a known history of MS. In this case, the patient’s clinical presentation was polyfocal but suggestive of a typical MS clinical relapse affecting cerebral hemispheres and infratentorial regions, making this case clinically challenging. Further, rapid progression after years of MS on fingolimod without relapsing episodes further supported a separate pathology from MS. In addition, an inadequate response to plasmapheresis and steroids with further expansion of the lesions on serial imaging shies away from a primary demyelinating process. The patient's repeat MRI showed the progression of multiple enhancing lesions that included a differential of lymphoreticular disease, metastatic disease, and PML. In general, mimics of MS can include various inflammatory, neoplastic, infectious, metabolic, and genetic disorders.

Both immunostaining and flow cytometry were positive for markers seen typically in B-cell lymphoma and negative for T-cell markers [6]. Further, testing also revealed a very high Ki-6, which is a reflection indicating the degree of tumor cell proliferation.

Another case report was found with a patient with a diagnosis of tumefactive MS [7]. The patient had been clinically and radiographically stable for three years on anti-CD20 monoclonal antibody ocrelizumab before presenting with headache, left-sided weakness, and increased fatigue. The patient was eventually diagnosed with diffuse large B-cell lymphoma, highlighting the fact that even while on therapy for MS, biopsy should be considered for new lesions seen on imaging if acute immunotherapies have no response to the patient’s clinical symptoms.

## Conclusions

It is important to keep a broad range of differential diagnoses in mind, given the wide variety of diseases that can mimic MS. Those with a known MS history should also have these differentials in mind. Each patient’s MS history should be taken into account when presenting with a possible relapse.
